# Potential Impact of Non-Steroidal Mineralocorticoid Receptor Antagonists in Cardiovascular Disease

**DOI:** 10.3390/ijms24031922

**Published:** 2023-01-18

**Authors:** Asadur Rahman, Nourin Jahan, Md Tanvir Rahman, Akira Nishiyama

**Affiliations:** 1 Department of Pharmacology, Faculty of Medicine, Kagawa University, 1750-1 Ikenobe, Miki-cho, Kita-gun, Kagawa 761-0793, Japan; 2 Department of Microbiology and Hygiene, Faculty of Veterinary Science, Bangladesh Agricultural University, Mymensingh 2202, Bangladesh

**Keywords:** cardiovascular disease, non-steroidal mineralocorticoid receptor antagonist, heart failure

## Abstract

Inappropriate mineralocorticoid receptor (MR) activation in different cardiovascular cell types has deleterious effects on cardiac remodeling and function. Therefore, MR inhibition is a crucial pharmacological strategy to overcome cardiovascular dysfunction. Despite efficient blockade of MR with steroidal MR antagonists (MRAs), their clinical application is unsatisfactory due to the adverse effects. Newer non-steroidal MRAs with greater potency could be suitable for clinical application, especially in patients with type 2 diabetes mellitus and chronic kidney disease. Although clinical evidence has shown the beneficial effects of non-steroidal MRAs on cardiovascular outcomes in patients with heart failure with reduced ejection fraction, clinical trials are ongoing to evaluate the efficacy of heart failure with preserved ejection fraction. Therefore, comparative pharmacological characterization of non-steroidal MRAs over classic steroidal MRAs is crucial. Here, we summarize the pre-clinical evidence of non-steroidal MRAs, which suggests an improvement in cardiac dysfunction, as well as the underlying molecular mechanisms in animal models mimicking different clinical conditions. In addition, we discuss up-to-date information from clinical trials regarding the beneficial effects of non-steroidal MRAs on meaningful cardiovascular outcomes. Both pre-clinical and clinical evidence support treatment with non-steroidal MRAs in patients with cardiovascular disease.

## 1. Introduction

Cardiovascular disease (CVD) is considered a global health concern, and its prevalence is increasing, especially in older people [1]. Among the different comorbidities of CVD, heart failure (HF) is a complex clinical condition that manifests as abnormal cardiac structure or function, thereby altering normal cardiac filling and the ejection of an appropriate blood volume to maintain peripheral organ perfusion. HF is defined according to the clinical measure of left ventricular (LV) ejection fraction (EF), which may either be reduced (<40%; HFrEF or systolic HF) or preserved (>50%; HFpEF or diastolic HF) [2,3]. Despite the enormous therapeutic advances that have emerged in the management of CVD in the past three decades, patients diagnosed with HF still have a very poor prognosis and quality of life. It is also the most common reason for hospitalization in the aged population [4]. Traditional guideline-directed therapies primarily slow the progression of cardiovascular disorders, but there is a need to develop novel, preventive, and reparative therapeutic strategies.

The mineralocorticoid receptor (MR) is expressed not only in epithelial cells in the distal nephron but also in other non-epithelial cells, including cardiomyocytes, cardiac fibroblasts, endothelial cells, and vascular smooth muscle (VSM) cells [5]. In addition to its critical role in the regulation of electrolyte and fluid homeostasis in the kidney, inappropriate MR activation has been shown to facilitate multiple deleterious actions in the cardiovascular system [6,7]. The classic steroidal MR antagonists (MRAs), spironolactone and eplerenone, have been validated to block the deleterious effects of MR activation, thereby exhibiting comprehensive cardiovascular protective actions. The cardiovascular endpoint trials RALES (Randomized ALdactone Evaluation Study) [8] and EMPHASIS-HF (Eplerenone in Mild Patients Hospitalization and Survival Study in Heart Failure) [9] conducted in patients with HFrEF, as well as ALDO-DHF (Aldosterone Receptor Blockade in Diastolic Heart Failure) [10] and TOPCAT (Treatment of Preserved Cardiac Function Heart Failure With an Aldosterone Antagonist) [11] conducted in patients with HFpEF, have demonstrated the beneficial effects of eplerenone or spironolactone on morbidity and mortality. However, the clinical use of available steroidal MRAs is restricted because of their major side effects, which include hyperkalemia, gynecomastia, impotence, and menstrual disturbances [12]. Recently, several non-steroidal MRAs have been developed and examined in subjects with CVD [13,14]. In this review, we briefly summarize and discuss the pre-clinical data in a comparative manner, focusing on the beneficial effects of non-steroidal and steroidal MRAs in different animal models with cardiac dysfunction. We also discuss the possible molecular mechanisms that underlie the protective effects of MRAs against cardiac remodeling and dysfunction. Moreover, we provide an update on cardiovascular endpoint outcomes with non-steroidal MRAs from different clinical studies.

The literature was gathered from online research databases, including NCBI, PubMed, and Google Scholar, using the keywords “mineralocorticoid receptor antagonist”, “steroidal MRA”, “nonsteroidal MRA”, “cardiovascular outcomes”, “eplerenone”, “spironolactone”, “finerenone”, and “esaxerenone”. The pre-clinical and clinical findings on the cardioprotective effects of MRAs from January 2000 to November 2022 are summarized in this review.

## 2. Chemical Properties of Steroidal and Non-Steroidal MRAs

The steroidal MRA, eplerenone, is around 40-fold less potent than spironolactone toward the MR; however, it exhibits much greater selectivity than spironolactone and shows less steroidal hormone-related adverse effects [12,15]. Among the recently developed non-steroidal MRAs, the cardiovascular endpoint outcomes of finerenone (BAY94-8862, Bayer) and esaxerenone (CS-3150, Daiichi Sankyo) have been published in both pre-clinical and clinical studies. Therefore, in this review, we mainly focused on these two non-steroidal MRAs. Finerenoene, which is a modified prototypical dihydropyridine derivative, is over 500-fold more selective for the MR and at least as potent as spironolactone [16,17]. Similarly, esaxerenone is 18-fold and 260-fold more potent than spironolactone and eplerenone, respectively, and at least 1400-fold more selective for the MR than other steroidal MRAs [18,19]. Taken together, non-steroidal MRAs can be stated to have greater affinity and suitable selectivity toward the MR over steroidal MRAs.

In terms of tissue distribution, steroidal MRAs have been suggested to likely accumulate in the kidney, which alters electrolyte homeostasis, leading to hyperkalemia [12,17,20]. In contrast, the non-steroidal MRAs finerenone and esaxerenone have a balanced distribution between the kidney and heart [21,22]. On the basis of the available information, non-steroidal MRAs appear to be potent and selective, and their tissue distribution and affinity are rather balanced in both the heart and kidney.

The pharmacological actions of different MRAs cause distinct regulation of MR target gene promoter activity based on ligand-specific gene regulation. The distinct clinical actions of different MRAs can be explained, at least in part, by the different binding modes to the MR and the initiation of MR modulation through different molecular mechanisms. Structurally different MRAs have different MR binding modes, resulting in distinct gene expression and ligand-specific clinical actions. The molecular mechanism of steroidal and non-steroidal MRAs is quite different, even though all of the MRAs bind to the same MR ligand-binding domain (MR-LBD). Spironolactone and eplerenone are both passive MRAs that are derivatives of aldosterone. However, spironolactone promotes co-factor (SRC-1) recruitment to an MR-dependent promoter, but to a much lesser extent than aldosterone [13,14]. Conversely, the non-steroidal MRA finerenone is a complete antagonist, unlike spironolactone and eplerenone, which binds to the ligand-binding domain of the MR, causing a conformational change inside the MR complex and thereby altering the stability, as well as causing nuclear translocation, of the MR [23]. Esaxerenone is an enantiomer of atropisomer that binds to the MR-LBD with large side chains, which are flipped and form a relatively large and secluded binding pocket [24]. Thus, the binding mode of non-steroidal MRAs is completely different to that of classic steroidal MRAs.

## 3. Pharmacological Effects of Steroidal and Non-Steroidal MRAs in CVD: Evidence from Pre-Clinical Studies

### 3.1. Blood Pressure

Hypertension is considered a predominant risk factor for the development and progression of CVD [25]. Steroidal MRAs have been proven to be effective in reducing blood pressure (BP), either alone or as add-on therapy. Similarly, non-steroidal MRAs have antihypertensive properties. Here, we explore the comparative effects of steroidal and non-steroidal MRAs on the reduction in BP in different animal models of CVD (Table 1).

Among the experimental animal models of CVD, hypertension-induced HF has been repeatedly studied in Dahl salt-sensitive (DSS) hypertensive rats with high salt (HS) loading. The steroidal MRAs eplerenone [19,26] and spironolactone [19] reduced the BP by around 8–10% compared with their counterparts in 8% salt diet-fed DSS rats at higher doses (100 mg/kg/day), but not at lower doses (10 and 30 mg/kg). Notably, the non-steroidal MRA esaxerenone caused a reduction in systolic BP (SBP) by approximately 18% and 27% at a dose of 1 and 2 mg/kg/day, respectively, in the same model (Table 1) [19]. However, HS loading for 6 weeks prior to esaxerenone treatment (1 mg/kg/day) for 4 weeks reduced the BP by around 9% [27]. The deoxycorticosterone acetate (DOCA)-salt model is frequently considered as a model of cardiovascular remodeling due to the persistently high BP. In this model in uninephrectomized (UNX) Wistar Kyoto rats, esaxerenone treatment (3 mg/kg) for 2 weeks significantly reduced the BP (30%), while eplerenone (30 mg/kg) and spironolactone (30 mg/kg) were not effective in reducing BP (3% decrease and 2% increase, respectively) [18]. Similarly, in the DOCA-salt model in Sprague–Dawley (SD) rats, the finerenone (10 mg/kg) treatment-induced reduction in BP was stronger than that of eplerenone (100 mg/kg) [21].

Finerenone also efficiently reduced the BP in nitrogen monoxide (nitric oxide) synthase inhibitor N(ω)-nitro-L-arginine methyl ester (L-NAME)-treated transgenic (mRen2)27 rats, which is a typical hypertensive model [28]. Moreover, in Munich Wistar Fromter rats with proteinuria and hypertension, finerenone treatment tended to reduce BP, even though the reduction was not statistically significant [29]. Importantly, in ovariectomized (OVX) mice, finerenone treatment caused a 13% reduction in BP compared with control [30].

All of the above information from pre-clinical studies suggests that non-steroidal MRAs effectively reduce BP in hypertensive animals. Notably, however, animal studies involve a large amount of drugs, which may not always be an accurate representation of the situation in clinical studies. Finerenone has not been clinically developed as an antihypertensive agent and is therefore not clinically used at BP-lowering doses.

**Table 1:** Effects of MRAs in different animal models and underlying molecular mechanism.

Salt-Induced Hypertensive Animal Models											
MRAs	Animal Model	Drug Dosage	Blood Pressure	Cardiac Remodeling		Cardiac Function	Vascular Changes	Molecular Mechanism			Ref.
Eplerenone	DSS rats; 8% NaCl diet for 12 weeks	10, 30, 100 mg/kg/day 7 weeks	⇩ SBP (8.5% for 100 mg/kg/day)	⇩ LVW/BW (32%, 39% and 44%) * ⇩ LVEDD (18%, 19%, 21%) * ⇧ LVES		⇧ % FS (20%, 22%, 25%) * ⇩ HR (13%, 9.6%, 13.2%) *	⇩ Wall:lumen ratio (38%, 46%, 43%) *	Oxidative stress	Fibrosis	Other	[26]
								⇩ NADPH oxidase activity (26%, 31%, 37% *) ⇩ p22phox ⇩ p47phox ⇩ gp91phox	⇩ Perivascular fibrosis (31%, 40%, 54% *) ⇧ SERCA2 ⇩ TGF-β1 ⇩ Collagen-I	⇧ P-eNOS ⇧ P-Akt ⇧ Nitrite production	
	DSS rats; 8% NaCl diet for 7 weeks	10, 30, 100 mg/kg/day 7 weeks	⇩ SBP (9% for 100 mg/kg/day)	⇩ LVW/BW (10%, 10%, 13%) * ⇨ BNP (8% ↓ for 100 mg/kg)		-	-	Other pathologies			[19]
								⇩ Urinary protein ⇩ Glomerulosclerosis (100 mg/kg) ⇨Tubular injury			
Spironolactone	DSS rats 8% NaCl diet for 7 weeks	10, 30, 100 mg/kg/day; 7 weeks	⇩ SBP (10% for 100 mg/kg/day)	⇩ LVW/BW (9%, and 12% for 30 and 100 mg/kg/day) ⇨ BNP (19% ↓ for 100 mg/kg)		-	-	Other pathologies			[19]
								⇨ Urinary protein excretion ⇩ Glomerulosclerosis (100 mg/kg)			
Esaxerenone	DSS rats 8% NaCl diet for 7 weeks	0.25, 0.5, 1 and 2 mg/kg; 7 weeks	⇩ SBP (6%, 12%, 18% and 27%) *	⇩ LVW/BW (13%, 19%, 26% for 0.5, 1 and 2 mg/kg/day, respectively) ⇩ BNP (38% and 43% ↓ for 1 and 2 mg/kg, respectively)		-	-	-			[19]
	DSS rats 8% NaCl diet for 10 weeks	1 mg/kg/day (0.001% esaxerenone *w*/*w*); 4 weeks	⇩ SBP (9%)	⇩ HW/BW (8%) ⇩ LVW/BW (8%) ⇩ NPPA ⇩ NPPB ⇩ MYH7 ⇩ LVIDs (13%) ⇩ ESV (33%)		⇧ EF (16%) ⇧ FS (25%) ⇧ SV (19%) ⇧ CO (26%) ⇨ HR	-	Oxidative stress	Inflammation	Fibrosis	[27]
								⇩ 4-HNE ⇩ gp47phox ⇩ p22phox ⇩ MDA	⇩ CXCL8 ⇩ TNF-α ⇩ IL-6	⇩ TGF-β ⇩ Collagen I and III ⇩ PAI-1 ⇩ SGK-1	
Finerenone	SD rats, UNX, DOCA/salt	0.1, 1, and 10 mg/kg/day; 10 weeks	⇩ SBP (10 mg/kg)	⇩ HW/BW (1 and 10 mg/kg) ⇩ pro-BNP (1 and 10 mg/kg) ⇩ Structural heart injury (10 mg/kg)			⇩ Focal vasculopathy (10 mg/kg)	- - ⇩ Fibrosis			[21]
Myocardial/Vascular Injury Model											
MRAs	Model	Drug Dosage	Blood Pressure	Cardiac Remodeling	Cardiac Function		Vascular Changes	Molecular Mechanism			Ref.
					Systolic	Diastolic		Oxidative Stress	Inflammation	Fibrosis	
Eplerenone	Wistar rats, coronary artery ligation (MI)	100 mg/kg/day	-	⇨ HW/BW ⇨ pro-BNP	⇨ dP/dt_max_ ⇨ dP/dt_min_ ⇨ HR	⇨ LVEDP ⇨ Tau	-	-	-	⇨ Osteopontin (mRNA)	[21]
Finerenone		0.1, 0.3, and 1 mg/kg/day, 8 weeks	-	⇨ HW/BW ⇩ pro-BNP	⇧ dP/dt_max_ ⇩ dP/dt_min_ (1 mg/kg) ⇨ HR	⇘ LVEDP ⇘ Tau (for 1 mg/kg)	-	-	-	⇩ Osteopontin (mRNA)	
Eplerenone	C57BL/6, transverse aortic constriction (TAC)	200 mg/kg/day, 4 weeks	-	⇨ LVM ⇨ IVS ⇨ LVPW ⇘ BNP ⇨ Tnnt2	⇨ EF ⇨ HR	-	-	-	-	⇨ Fibrosis	[31]
Finerenone		10 mg/kg/day, 4 weeks	-	⇩ LVM ⇩ IVS ⇩ LVPW ⇩ BNP ⇩ Tnnt2 (mRNA)	⇨ EF ⇩ HR	-	-	-	-	⇨ Fibrosis	
Finerenone	C57BL/6, artery injury model	1 and 10 mg/kg/day, 10 days	-	-	-	-	⇧ Re-endothelialization ⇩ Ki-67 ⇩ α-SMA ⇩ Neointimal lesion formation	-	⇩ CD45	-	[32]
Eplerenone	129/Sv mice, isoproterenol 25 mg/kg for 4 days	200 mg/kg/day	⇩ SBP (12.5%)	⇩ HW/TL ⇩ LVM/TL ⇩ GLS	⇘ EF ⇨ ESV	⇨ EDV ⇩ LVAWd ⇩ LVPWd	-	⇩ NADPH oxidase 1	⇨ CD 68	⇘ TNX ⇩ TGF-β ⇩ Col1a1 ⇘ Gal3	[33]
Finerenone		10 mg/kg/day	⇘ SBP (6.7%)	⇩ HW/TL ⇩ LVM/TL ⇩ GLS	⇘ EF ⇩ ESV	⇨ EDV ⇩ LVAWd ⇩ LVPWd	-	⇩NADPH oxidase 1	⇩ CD 68	⇩ TNX ⇩ TGF-β ⇩ Col1a1 ⇩ Gal3	
Transgenic Hypertensive Model											
MRAs	Model	Drug Dosage	Blood Pressure	Cardiac Remodeling	Cardiac Function		Vascular Changes	Molecular Mechanism			Ref.
Finerenone	Renin-transgenic (mRen2)27 rats, L-NAME	1 and 3 mg/kg; 7 weeks	⇩ SBP	⇨ Pro-BNP	-		⇘ Vasculopathy ⇘ Vascular fibrosis	⇘ Fibrosis			[28]
	RacET transgenic mice	100 ppm with chow; 5 months	-	⇨ HW ⇨ LVW ⇩ LAW ⇩ LA/LV	**Systolic**	**Diastolic**	-	**Oxidative stress**	**Fibrosis**	**Other**	[34]
					⇨ FS ⇗ EF ⇨ LVDs ⇘ LVESV ⇘ SV	⇨ LVDs ⇘ LVEDV ⇨ E/e′ ratio		⇩ NADPH oxidase activity	⇩ TGF-β ⇩ CTGF ⇩ LOX ⇩ Osteopontin ⇩ COL3A1	⇩ MR nuclear translocation	
	MWF rats	10 mg/kg/day; 4 weeks	⇩ SBP	-	-	-	⇧ Ach-induced relaxation ⇩ NA-induced constriction ⇩ AngII-induced constriction	⇧ p47phox ⇧ Mn-SOD ⇧ Cu/Zn-SOD ⇧ P-eNOS	-	⇩ UAE	[29]
Metabolic Syndrome Model											
MRAs	Model	Drug Dosage	Blood Pressure	Cardiac Remodeling	Cardiac Function		Vascular Changes	Molecular mechanism			Ref.
					Systolic	Diastolic		Oxidative Stress	Inflammation	Fibrosis	
Spironolactone	C57BL6J (female), Western diet with high fat	1 mg/kg/day; 7 days	⇨ SBP ⇨ DBP ⇨ MAP	⇩ LV weight ⇧ sarcomere lengths	⇩ LVDs ⇨ LVESP ⇨ LVESPVR	⇧ *E′*/*A′* ⇨ *E*/*e′* ⇩ IVRT ⇩ Diastolic relaxation time	-	⇩ ROS	⇩ M1 MΦ ⇧ M2 MΦ	⇨ Interstitial fibrosis	[35]
Finerenone	Zucker fatty fa/fa rats	2 mg/kg; 7 days	⇨ SBP	⇨ LVW ⇧ LV tissue perfusion	⇩ LVDs ⇨ LVESP ⇨ LVESPVR ⇧ FS	⇨ LVDd ⇘ LVEDP ⇩ LVEDPVR ⇩ Tau	⇨ TPR	**Oxidative stress**	**Fibrosis**	**Other**	[36]
								⇩ LV ROS ⇩ Plasma nitrite	⇨ Collagen	⇘ Proteinuria	
		2 mg/kg; 90 days	⇨ SBP	⇩ LVW ⇧LV tissue perfusion	⇩ LVDs ⇨ LVESP ⇨ LVESPVR ⇧ FS	⇩ LVDd ⇩ LVEDP ⇩ LVEDPVR ⇘ Tau	⇨ TPR	-	⇩ Collagen	⇩ Proteinuria	
Finerenone	OVX (at 4 month)	1 mg/kg/day (from 7 months); 1 month	⇩ SBP (13%) ⇨ DBP	⇨ HW/TL ⇨ LVW/TL ⇨ MCSA ⇨ Capillary density	⇨ LVESP ⇨ LVESPVR ⇨ dP/dt_min_ ⇨ FS ⇨ CO	⇩ LVEDP ⇩ LVEDPVR ⇨ Tau	⇧ Vascular relaxation	**Oxidative stress**	**Fibrosis**	**Inflammation**	[30]
								⇨ LV ROS ⇨ IFM ROS ⇨ SSM ROS	⇨ Col-I ⇨ Col-III	PECAM-1+ vessels	
Nephrectomy Model											
MRAs	Model	Drug Dosage	Blood Pressure	Cardiac Remodeling		Cardiac Function		Vascular Changes	Molecular Mechanism		Ref.
						**Systolic**	**Diastolic**				
Finerenone	UNX+ DOCA/salt	0.1, 1 and 10 mg/kg/day; 10 weeks	⇩ SBP (10 mg/kg)	⇩ HW/BW ⇩ pro-BNP (1 and 10 mg) ⇩ Structural heart injury (10 mg/kg)		-	-	⇩ Focal vasculopathy (10 mg/kg)	⇩ Fibrosis		[21]
Finerenone	Subtotal Nx; B6D2 male	2.5 mg/kg/d with chow; 6 weeks	⇨ SBP ⇩ DBP	⇩ HW/TL ⇨ Cardiomyocytes cross-sectional area ⇘ nppb (BNP)		⇗ HR ⇗ EF ⇧ FS ⇨ SV ⇨ CO	⇧ E/A ratio ⇨ dP/dtmax ⇨ dP/dtmin ⇨ Tau	-	⇘ Interstitial fibrosis ⇩ α-SMA ⇧ NOV		[37]

DSS rat, Dahl salt-sensitive rat; SBP, systolic blood pressure, LVW, left ventricular weight; BW, body weight, LVEDD, left ventricular end-diastolic diameter; LVES, left ventricular end-systolic; FS, fractional shortening; HR, heart rate; NADPH, nicotinamide adenine dinucleotide phosphate; SERCA2, sarco/endoplasmic reticulum Ca^2+^-ATPase 2; TGF-β1, transforming growth factor-β1; P-eNOS, phospho nitric oxide synthase; BNP, brain or b-type natriuretic peptide; NPPA, encodes atrial natriuretic peptide (ANP); NPPB, encodes BNP; MYH7, encodes β-myosin heavy chain; LVID, left ventricular internal diameter; ESV, end-systolic volume; EF, ejection fraction; SV, stroke volume; CO, cardiac output; 4-HNE, 4-hydroxynonenal; MDA, malondialdehyde; CXCL8, C-X-C motif chemokine ligand 8; TNF-α, tumor necrosis factor-α; IL-6, interleukin-6; PAI-1, plasminogen activator inhibitor-1; SGK-1, serum/glucocorticoid-regulated kinase 1; dP/dt_max_, maximal rate of rise of left ventricular pressure (LVP); dP/dt_min_, maximal rate of rise of LVP; LVEDP, left ventricular end-diastolic pressure; LVEDD, LV end-diastolic diameter; LVESD, LV end-systolic diameter; Tau, left ventricular diastolic time constant; IVS, interventricular septum; LVPW, left ventricular posterior wall; Tnnt2, encodes cardiac troponin T; Ki-67, proliferation marker; α-SMA; α-smooth muscle actin; CD45, cluster of differentiation 45; GLS, global longitudinal strain; LVM/TL, left ventricular mass/tibial length; EDV, end-diastolic volume; LVAWd, left ventricular anterio wall diameter; LVPWd, left ventricular posterior wall diameter; TNX, tenascin-X; Gal3, galectin-3; LAW, left atrial weight; E/e’ ratio, index for LV filling pressure; CTGF, connective tissue growth factor; LOX, lysyl oxidase; DBP, diastolic blood pressure; MAP, mean arterial pressure; LVESPVR, left ventricular end-systolic pressure-volume relations; IVRT, isovolumic relaxation time; MΦ, macrophage; LVEDPVR, left ventricular end-diastolic pressure-volume relations; TPR, total peripheral resistance; OVX, ovariectomy; ROS, reactive oxygen species, MCSA, maximal cross-sectional area; IFM, interfibrillar mitochondria; SSM, subsarcolemmal mitochondria; UNX, uninephrectomy; Nx, nephrectomy; NOV, nephroblastoma overexpressed; ⇧, increased; ⇩, decreased; ⇨, not changed, ⇗, tended to be increased; ⇘, tended to be decreased; *, dose-dependently.

### 3.2. Myocardial Structural Remodeling

During CVD progression, complex alterations in myocardial structure are commonly associated with cardiomyocyte apoptosis and interstitial and perivascular collagen fiber accumulation [38]. MRAs have consistently shown beneficial effects on pathological myocardial remodeling [39]. Here, evidence from pre-clinical studies is discussed to compare the beneficial effects of steroidal and non-steroidal MRAs on myocardial remodeling (Table 1).

In HS-loaded DSS rats, LV weight and LV end-diastolic diameter were significantly reduced, and LV end-systolic elastance was increased, in response to eplerenone, while BP was not altered (10 mg/kg) [26]. Spironolactone reduced LV weight at a higher dose (30 mg/kg) [19]. In contrast, a significant reduction in LV weight and LV internal diameter was evident with esaxerenone at doses as low as 0.5–1 mg/kg [19]. In the same study, the plasma concentration of brain natriuretic peptide (BNP), which is a well-accepted biomarker of cardiac remodeling, was not significantly reduced, even with a high dose of eplerenone (100 mg/kg). However, it was significantly reduced by esaxerenone at a dose as low as 1 mg/kg in salt-loaded DSS rats [19]. In the DOCA/salt model, finerenone caused a significant reduction in heart weight by 1 mg/kg, but structural heart injury was reduced by 10 mg/kg [21]. Moreover, in the same study, the reduction in BNP concentration with 1 mg/kg finerenone was comparable to that of 100 mg/kg eplerenone.

In a rat model of myocardial infarction with coronary artery ligation, neither eplerenone (100 mg/kg) nor finerenone (1 mg/kg) reduced the heart weight compared with their control counterparts. However, the plasma concentration of pro-BNP was significantly reduced only in finerenone-treated rats [21]. In the pressure overload-induced cardiac hypertrophy model in mice with transverse aortic constriction (TAC), the LV mass (normalized to body weight), interventricular septum thickness, and LV posterior wall thickness were significantly reduced by finerenone treatment, while eplerenone did not change these parameters [31]. The mRNA expression of BNP was reduced, both in the eplerenone and finerenone treatment groups, compared with their control counterparts. However, troponin T type 2, which is a crucial contractile protein, was significantly downregulated by finerenone, but not eplerenone. Another study showed that finerenone treatment caused a significant reduction in interventricular septal and LV posterior wall thickness [31]. Transgenic mice with cardiac-specific overexpression of Ras-related C3 botulinum toxin substrate 1 (Rac1) showed increased LV end-systolic and diastolic volumes, as well as left atrial (LA) weight, compared with wild-type control mice. Treatment with finerenone for 5 months prevented LV dilatation and significantly reduced LA weight [34]. TAC induced cardiac LV hypertrophy in mice [40], but treatment with finerenone, but not eplerenone, reduced the LV mass in association with a reduction in the interventricular septal thickness in diastole and LV posterior wall thickness in systole [31]. In mice with isoproterenol-induced cardiac hypertrophy, the equi-natriuretic dose of eplerenone and finerenone caused a reduction in the heart weight, LV mass, LV anterior wall thickness in diastole, LV posterior wall thickness in diastole, and global longitudinal peak strain, which is a marker of tissue deformation in the longitudinal direction of the heart; however, finerenone exhibited greater efficacy than eplerenone [33].

All of the above pre-clinical studies show that both steroidal and non-steroidal MRAs have beneficial effects on adverse cardiac remodeling, but non-steroidal MRAs have relatively stronger efficacy than their steroidal counterparts. 

### 3.3. Myocardial Function

In a previous study, salt-loaded DSS rats exhibited hypertensive HF due to volume overload [41]. Kobayashi et al. [26] demonstrated that LV percent fractional shortening (%FS), which is an index of systolic function, was significantly reduced with HS loading for 12 weeks in DSS rats, while treatment with eplerenone attenuated these changes independent of changes in BP. In the same animal model, HS loading for 10 weeks caused systolic dysfunction with an increase in LV end-systolic diameter (LVIDs) and a reduction in EF, %FS, stroke volume (SV), and cardiac output (CO) [27]. Esaxerenone treatment (1 mg/kg) significantly improved systolic dysfunction with a 13% reduction in LVIDs and a significant increase in EF (16%), %FS (25%), SV (21%), and CO (26%) [27]. These data indicate that the non-steroidal MRA esaxerenone has cardioprotective effects in salt-sensitive hypertensive animals.

The ischemia-driven chronic myocardial infarction model induces both systolic and diastolic dysfunction. An alteration in myocardial contractility and relaxation was evidenced by a decrease in dp/dtmax and a deterioration in dp/dtmin following 8 weeks of myocardial infarction in rats. These characteristic parameters of LV systolic function in HF were significantly ameliorated by finerenone (1 mg/kg) but not eplerenone (100 mg/kg). In contrast, LV end-diastolic pressure and LV relaxation time constant (tau), which are indices of diastolic function, were significantly increased in mice with myocardial infarction. In the case of finerenone treatment (1 mg/kg), a decreasing trend was obvious for these parameters of LV diastolic dysfunction; however, there was no observable change in the case of eplerenone (100 mg/kg). In mice with TAC-induced LV hypertrophy, altered cardiac remodeling was improved by finerenone; however, cardiac function was not significantly changed either by TAC intervention or by finerenone/eplerenone treatment. Above all, the TAC-induced increase in heart rate was reduced by finerenone but not eplerenone [31].

Transgenic mice with myocardial-specific overexpression of constitutive active Rac1 (RacET) showed increased LV end-diastolic and end-systolic volumes, while treatment with finerenone normalized these parameters [34]. These data suggest that finerenone prevented MR-mediated structural remodeling in RacET mice.

OVX mice exhibit isolated diastolic dysfunction without any reduction in LV EF. Consistently, Pieronne-Deperrois et al. [30] demonstrated that the OVX-induced alteration in diastolic dysfunction was completely attenuated by finerenone, as evidenced by the improvement in LV filling pressure, as well as LV diastolic compliance. In Zucker fatty rats, which are used as a model of metabolic syndrome-associated cardiorenal injury, short-term (7 days) finerenone treatment caused a reduction in LV systolic diameter, resulting in an increase in LV FS and a reduction in the LV end-diastolic pressure-volume relationship and the LV relaxation time constant Tau, while it tended to reduce LV end-diastolic pressure and increase LV tissue perfusion [36]. However, in case of long-term (90 days) intervention with finerenone, a reduction in LV diastolic diameter was evident. Subtotal nephrectomy-associated renal failure induced a moderate decrease in LV FS and EF, but these alterations were fully prevented with finerenone [37]. Remarkably, these mice exhibited diastolic dysfunction because there was a substantial decrease in the trans-mitral pulse Doppler E/A ratio (a ratio of the early to late ventricular filling velocities), but treatment with finerenone increased the E/A ratio. Collectively, these data indicate that the non-steroidal MRA finerenone has the potential to improve both systolic and diastolic dysfunction in various rodent models.

### 3.4. Vascular Remodeling and Function

The MR is expressed in vascular endothelial and VSM cells, and it is crucial for the contractile and relaxation responses of the vasculature. Genetic inactivation of the MR in endothelial cells ameliorated DOCA/salt-induced cardiac remodeling in a previous study [42]. Furthermore, finerenone treatment accelerated re-endothelialization, as well as attenuated VSM cell proliferation and neointimal lesion formation, reducing inflammation and exerting a protective effect against vascular remodeling in C57BL/6 mice [32]. Pharmacological blockade of the MR with finerenone reduced focal vasculopathy and vascular fibrosis in DOCA/salt-loaded UNX SD rats [21] and L-NAME-treated (mRen2)27 transgenic rats [28]. Intervention with finerenone enhanced acetylcholine-induced relaxation in the coronary artery in OVX mice [30], while it reduced the noradrenaline and angiotensin II-induced contractile response in the thoracic artery [29]. These data indicate that blockade of the MR with non-steroidal MRAs attenuates vascular injury during CVD development.

## 4. Molecular Mechanisms Underlying the Beneficial Effects of Non-Steroidal MRAs on CVD: Evidence from Pre-Clinical Studies

### 4.1. Oxidative Stress

Cumulative evidence demonstrates the impact of MR activation and associated oxidative stress in cardiac tissues, which contribute to the development of LV remodeling and dysfunction [43,44]. Of note, activation of the MR in a ligand-dependent manner positively regulates nicotinamide adenine dinucleotide phosphate (NADPH) oxidase subunits, an important component for reactive oxygen species (ROS) generation [45]. HS-loaded DSS rats exhibited high expression of 4-hydroxynonenal, increased expression of the components of NADPH oxidase gp47phox and p22phox, and elevated lipid peroxidation, as evidenced by an increased level of malondialdehyde in cardiac tissues (Table 1) [27]. However, esaxerenone treatment significantly improved all of these indicators of oxidative stress [27]. In contrast, ligand-independent activation of the MR is associated with ROS generation through the Rac1-dependent pathway [46]. Importantly, finerenone treatment attenuated NADPH oxidase activity in RacET transgenic mice with constitutively active Rac1 in the myocardium [34]. In Zucker fatty rats, short-term treatment with finerenone caused a reduction in LV ROS production and an increase in the bioavailability of nitric oxide, as evidenced by an increased plasma nitrite concentration [36]. In OVX mice, finerenone efficiently reduced superoxide-mediated endothelial dysfunction in the coronary arteries [30].

### 4.2. Inflammation

Cardiomyocyte-specific MR knockdown leads to a reduced inflammatory burden in abdominal aortic constriction in mice [47] and an L-NAME-treated hypertensive animal model [48], as well as a dramatic decrease in the infiltration of inflammatory cells in DOCA/salt-loaded mice [49]. In contrast, activation of the MR contributed to the maintenance of inflammation through activation of the nuclear factor kappa B (NF-κB) pathway, as well as the initiation of pro-inflammatory cytokine expression [50]. These data suggest the critical role of the MR in CV remodeling, dysfunction, and target end-organ damage. HS-loaded DSS rats exhibited very high levels of CXCL8, which is a key regulator of inflammatory cell influx, while esaxerenone treatment downregulated the abundance of CXCL8 in ventricular tissues. Additionally, inflammatory cytokines, specifically tumor necrosis factor (TNF)-α and interleukin (IL)-6, were reduced by esaxerenone treatment [27]. In a wire-induced femoral artery injury mouse model, finerenone significantly reduced intimal and medial leukocyte content, as evidenced by a decrease in the abundance of CD45+ cells [32]. Moreover, activation of the macrophage MR modulated their status toward the pro-inflammatory M1 phenotype, while MR knockdown in macrophages induced an anti-inflammatory M2 phenotype [48]. No evidence of macrophage polarization in response to non-steroidal MRAs in ventricular tissues has been established. However, in the ischemic kidney, finerenone led to a 50% reduction in TNF-α and IL-1β (M1 markers) and a significant increase in plasminogen activator inhibitor (PAI)-1, mannose receptor, peroxisome proliferator-activated receptor-γ, IL-10, and arginase 1 (M2 markers) [51]. These data indicate that non-steroidal MRAs could be equipotent to the steroidal MRA eplerenone [52] in terms of enhancing cardiac macrophage polarization toward the M2 phenotype. Other than the contribution to innate immunity, the MR contributes significantly to adaptive immune cells, specifically in T lymphocytes, which play an important role in cardiovascular remodeling [47]. In DOCA/salt-loaded animals, MR activation in dendritic cells led to T-cell polarization toward the pro-inflammatory (Th1/Th17) phenotype [53]. However, finerenone treatment attenuated cardiorenal injury by reducing renal RORγt γδ T-cell populations [54]. Transcriptome analysis in human kidney cells revealed that finerenone exhibited efficient antagonism of aldosterone-induced or aldosterone-suppressed genes [55]. During renal fibrosis progression, aldosterone represses pro-inflammatory markers (IL-6, IL-11, CCL7, and CXCL8), while finerenone prevents this repression. However, future studies should be undertaken to understand the effects of non-steroidal MRAs in the regulation of cardiac inflammation.

### 4.3. Interstitial Fibrosis

Cardiac fibrosis is caused by elevated accumulation and cross-linking of extracellular matrix proteins, leading to increased myocyte stiffening, which can impair ventricular relaxation and contraction of the myocardium [56]. Numerous studies have shown the critical role of MR activation in diffuse interstitial and perivascular collagen deposition in the process of cardiac fibrosis [57,58]. Although steroidal MRAs were effective in reducing cardiac fibrosis [26], in this study, we focused on the molecular mechanism responsible for the protective effects of non-steroidal MRAs on cardiac fibrosis.

HS-loaded DSS rats exhibited interstitial and perivascular fibrosis in LV tissues. Treatment with esaxerenone reduced cardiac fibrosis in association with a reduction in transforming growth factor (TGF)-β, which is a pleiotropic mediator of cardiac fibrosis; collagen type I and III, which are major extracellular matrix proteins; and PAI-1, which is an inducer of regional cytokine production [27]. Serum and glucocorticoid-regulated kinase (SGK)-1 plays an important role in angiotensin or MR-induced cardiac fibrosis [59]. In HS-loaded DSS rats, esaxerenone treatment blunted the elevation in SGK-1 [27]. In neonatal rat cardiac fibroblasts, finerenone treatment attenuated aldosterone-induced nuclear MR translocation in association with the downregulation in connective tissue growth factor (CTGF), which is a central pro-fibrotic mediator that induces collagen production and subsequent pro-fibrotic enzymes; lysyl oxidase (LOX), which is a mediator of collagen cross-linking to form stable collagen fibers; TGF-β; and fibronectin, which is an extracellular matrix protein that regulates proliferation, differentiation, migration, and adhesion (Table 2) [34]. 

Consistently, finerenone in transgenic mice with cardiac-specific overexpression of Rac1 led to the downregulation of TGF-β, CTGF, LOX, osteopontin (a pro-fibrotic marker), and collagen 3α1 [34]. In the Zucker fatty metabolic syndrome animal model [36], as well as in OVX rats [30], finerenone significantly reduced collagen deposition in LV tissue. In the rat model of chronic myocardial infarction, finerenone-induced improvements in systolic and diastolic function were associated with a dose-dependent decrease in the mRNA expression of osteopontin [21]. Moreover, in a mouse model of subtotal nephrectomy, finerenone reduced interstitial fibrosis in association with attenuating the increased expression of α-smooth muscle actin, a fibroblast activation marker, and caused increased expression of nephroblastoma, an anti-fibrotic cytokine [37].

### 4.4. Vascular Injury

Aggravated activation of the MR in vascular endothelial and VSM cells has been indicated to contribute to the pathophysiology of HF [60]. Recent in vitro studies have demonstrated the preventive effects of finerenone in aldosterone-induced human umbilical vein endothelial cell apoptosis and coronary artery smooth muscle cell proliferation (Table 2) [32]. Furthermore, endothelial dysfunction in the coronary arteries is efficiently reduced by treatment with finerenone in OVX mice [30]. Aldosterone promotes atherosclerosis [61], where low-density lipoprotein cholesterol (LDL)-C plays a central role [62]. However, finerenone treatment reduces intrinsic arterial stiffness in Munich Wistar Frömter (MWF) rats accompanied by changes in elastin organization, normalization of matrix metalloproteinases, and reduction of oxidative stress [63]. Taken together, non-steroidal MRAs, especially finerenone, may have a protective role against vascular injury.

## 5. Pharmacological Effects of Non-Steroidal MRAs on Cardiovascular Outcomes: Evidence from Clinical Studies

In a retrospective study in hypertensive patients with HFrEF treated with non-steroidal MRAs, esaxerenone was proven to be safe without inducing hyperkalemia and hypotension [64]. In addition, nocturnal hypertension is considered an important marker of CVD [65,66], and Kario et al. [67] demonstrated that esaxerenone has suitable BP-lowering efficacy in patients with nocturnal hypertension.

Among the data from phase 2 studies using finerenone have been available, but three clinical trials (ARTS (minerAlocorticoid Receptor Antagonist Tolerability Study) [68], ARTS-HF [69], and ARTS-HF Japan [70]) have investigated cardiovascular outcomes in patients with HFrEF with chronic kidney disease (CKD) or diabetes mellitus. In the ARTS trial, in patients with HFrEF and moderate CKD, finerenone caused a smaller mean increase in serum potassium compared with spironolactone and decreased N-terminal pro-BNP to a similar degree as spironolactone [68]. Of note, finerenone was associated with a lower incidence of hyperkalemia and worsening renal function. Moreover, in the ARTS-HF study in which finerenone treatment was given in a dose-escalation manner in patients with worsening HFrEF and CKD and/or diabetes mellitus, treatment with finerenone and eplerenone induced a 30% or greater decrease in the N-terminal pro-BNP concentration in a similar proportion of patients [69]. With the same primary endpoint in the ARTS-HF Japan study [70], finerenone was well tolerated in Japanese patients, while the serum potassium concentration was similar between the finerenone and eplerenone treatment groups. In addition to these phase 2 studies, two phase 3 studies (FIDELIO-DKD [Finerenone in Reducing Kidney Failure and Disease Progression in Diabetic Kidney Disease] [71] and FIGARO-DKD [Finerenone in Reducing Cardiovascular Mortality and Morbidity in Diabetic Kidney Disease] [72]) have been completed. In the FIDELIO-DKD study, the primary endpoints were associated with kidney outcomes, but the secondary outcomes were associated with cardiovascular death, non-fatal myocardial infarction, non-fatal stroke, or hospitalization for HF. In patients with CKD and type 2 diabetes mellitus, finerenone treatment was associated with a lower risk of cardiovascular events than placebo, with the exception of non-fatal stroke, which was similar between the groups [71]. Cardiac remodeling and kidney complications enhanced the risk of atrial fibrillation or flutter (AFF) in patients with CKD and diabetes mellitus [73,74]. In the FIDELIO-DKD study [75], finerenone reduced the risk of new-onset AFF, as well as the risk of cardiovascular events, irrespective of the history of AFF at baseline. Then, the primary outcome, which was a composite of death from cardiovascular causes, non-fatal myocardial infarction, non-fatal stroke, or hospitalization for HF, was set in the FIGARO-DKD study [72]. In the finerenone treatment group, primary outcome events occurred in 12.4% of patients compared with 14.2% in the placebo group in patients with type 2 diabetes mellitus with CKD. These data suggest a definitive improvement in cardiovascular outcomes with finerenone. A new phase 3 study FINEARTS-HF (NCT04435626) with finerenone, is ongoing with the primary outcomes of the number of cardiovascular deaths and HF events in patients with HF and an LVEF of ≥40% (HFpEF). A phase 3 clinical trial with esaxerenone demonstrated BP reduction in patients with CKD and type 2 diabetes mellitus without available information about the cardiovascular composite outcome [76].

## 6. Conclusions

Despite the availability of advanced therapies, the composite outcomes for a cardiovascular endpoint in patients with HF are not satisfactory. Inappropriate overactivation of the MR induces inflammation and fibrosis, both of which cause adverse cardiac remodeling, cardiac dysfunction, and end-organ damage in patients with HF. Although treatment with classic steroidal MRAs has consistently shown an improvement in cardiovascular outcomes, their clinical applications are limited due to their adverse effects. Several non-steroidal MRAs have been developed with improved potency and selectivity over steroidal MRAs. Here, we have discussed the pre-clinical evidence of non-steroidal MRAs and possible molecular mechanisms underlying cardioprotective effects elaborately and summarized the information in Table 1 and Table 2. Due to the convincing therapeutic efficacies, especially in terms of cardioprotective effects, we have mainly discussed finerenone and esaxerenone among the available non-steroidal MRAs.

The non-steroidal MRAs differ in terms of not only their chemical structures but also their selectivity and potency to block the MR, their distribution in the heart and kidneys, and their molecular mechanisms of action. Clinical trials on cardiovascular composite endpoint outcomes are available only for finerenone. The results from the FIDELIO-DKD and FIGARO-DKD studies are optimistic and support a paradigm shift in the management of CVD using non-steroidal MRAs in patients with HFrEF. Current therapies for patients with HFpEF are limited. Pre-clinical data have shown that finerenone can improve both systolic and diastolic cardiac function (Table 1). However, future studies should be undertaken in different clinical conditions for better prediction. We hope that promising data will soon be available from an ongoing clinical trial (FINEARTS-HF) with finerenone in patients.

## Figures and Tables

**Table 2:** Effects of MRAs in different cells and underlying molecular mechanism.

In Vitro Model				
MRAs	Cells/Model	Drug Dosage	Molecular Mechanism	Ref.
Eplerenone	H9C2/MR+ cardiomyocytes, aldosterone 1, 10, 100 nmol/L	0.05, 0.5, 5, 50 µM	⇩ TNX ⇩ Adamts-1	[33]
Finerenone		0.05, 0.5, 5 µM	⇩ TNX ⇩ Adamts-1	
Finerenone	Neonatal cardiac fibroblast; aldosterone 10 nM, 24 h/Ang II 1 µM, 3 h	500 nm for 25 h	⇩ CTGF ⇩ TGF-β ⇩ LOX ⇩ miR-21 ⇩ Fibronectin ⇩ MR nuclear translocation	[34]
Finerenone	Human coronary artery smooth muscle cells, aldosterone 10, 20, 50 nM, 24 h	1 and 10 nM	⇩ Proliferation ⇘ Apoptosis	[32]
	Human umbilical vein endothelial cells, aldosterone 10, 20, 50 nM, 24 h		⇩ Proliferation ⇩Apoptosis	

Adamts-1, a disintegrin and metallopeptidase with thrombospondin type 1 motif 1; miR-21, microRNA-21; ⇩, decreased; ⇘, tended to be decreased.

## Data Availability

Not applicable.
